# Two explicit formulas for the generalized Motzkin numbers

**DOI:** 10.1186/s13660-017-1313-3

**Published:** 2017-02-15

**Authors:** Jiao-Lian Zhao, Feng Qi

**Affiliations:** 1 State Key Laboratory of Integrated Service Networks, Xidian University, Xi’an, Shaanxi 710071 China; 2 Department of Mathematics and Physics, Weinan Normal University, Weinan, Shaanxi 714009 China; 3 Department of Mathematics, College of Science, Tianjin Polytechnic University, Tianjin, 300387 China; 4 Institute of Mathematics, Henan Polytechnic University, Jiaozuo, Henan 454010 China

**Keywords:** 05A15, 05A19, 05A20, 05A15, 11B37, 11B83, explicit formula, Motzkin number, generalized Motzkin number, restricted hexagonal number, Catalan number, generating function, Faà di Bruno formula, Bell polynomial of the second kind

## Abstract

In the paper, by the Faà di Bruno formula, the authors establish two explicit formulas for the Motzkin numbers, the generalized Motzkin numbers, and the restricted hexagonal numbers.

## Introduction and main results

The Motzkin numbers $M_{n}$ enumerate various combinatorial objects. In 1977, fourteen different manifestations of the Motzkin numbers $M_{n}$ were given in [1]. In particular, the Motzkin numbers $M_{n}$ give the numbers of paths from $(0,0)$ to $(n,0)$ which never dip below the *x*-axis $y=0$ and are made up only of the steps $(1,0)$, $(1,1)$, and $(1,-1)$.

The first seven Motzkin numbers $M_{n}$ for $0\le n\le6$ are $1, 1, 2, 4, 9, 21, 51$. All the Motzkin numbers $M_{n}$ can be generated by

1.1 $$ M(x)=\frac{1-x-\sqrt{1-2x-3x^{2}} }{2x^{2}} =\frac{1}{1-x+\sqrt{1-2x-3x^{2}} } =\sum _{k=0}^{\infty}M_{k}x^{k}. $$

They can be connected with the Catalan numbers

$$ C_{n}=\frac{1}{n+1}\binom{2n}{n} $$

by

$$ M_{n}=\sum_{k=0}^{\lfloor n/2\rfloor} \binom{n}{2k}C_{k} \quad\mbox{and}\quad C_{n+1}=\sum _{k=0}^{n}\binom{n}{k}M_{k}, $$

where $\lfloor x\rfloor$ denotes the floor function whose value is the largest integer less than or equal to *x*. For detailed information, please refer to [2] and the closely related references therein. For information on many results, applications, and generalizations of the Catalan numbers $C_{n}$, please refer to the monographs [3, 4], the papers [5–13], the survey article [14], and the closely related references therein.

In [15], the $(u,l,d)$-Motzkin numbers $m_{n}^{(u,l,d)}$ were introduced and it was shown in [15], Theorem 2.1, that $m_{n}^{(u,l,d)}=m_{n}^{(1,l,ud)}$,

1.2 $$ M_{u,l,d}(x)=\frac{1-lx-\sqrt{(1-lx)^{2}-4udx^{2}} }{2udx^{2}}=\sum _{n=0}^{\infty}m_{n}^{(u,l,d)}x^{n}, $$

and

1.3 $$ m_{n}^{(u,l,d)}=l^{n}\sum _{j=0}^{n/2}\frac{1}{j+1}\binom{2j}{j} \binom {n}{2j} \biggl(\frac{ud}{l^{2}} \biggr)^{j}. $$

Comparing (1.1) with (1.2) reveals that $m_{n}^{(1,1,1)}=M_{n}$ and the $(u,l,d)$-Motzkin numbers $m_{n}^{(u,l,d)}$ generalize the Motzkin numbers $M_{n}$.

In [16], the Motzkin numbers $M_{n}$ were generalized in terms of the Catalan numbers $C_{n}$ to

$$ M_{n}(a,b)=a^{n}\sum_{k=0}^{\lfloor n/2\rfloor} \binom{n}{2k} \biggl(\frac {b}{a^{2}} \biggr)^{k}C_{k} $$

for $a,b\in\mathbb{N}$ and the generating function

1.4 $$ M_{a,b}(x)=\frac{1-ax-\sqrt{(1-ax)^{2}-4bx^{2}} }{2bx^{2}} =\sum _{k=0}^{\infty}M_{k}(a,b)x^{k} $$

was discovered. It was pointed out in [2] that

1.5 $$ M_{n}(1,1)=M_{n}, \qquad M_{n}(2,1)=C_{n+1}, \quad\mbox{and} \quad M_{n}(3,1)=H_{n}, $$

where $H_{n}$ denote the restricted hexagonal numbers and were described in [17].

For more information on many results, applications, and generalizations of the Motzkin numbers $M_{n}$, please refer to [1, 2, 16, 18, 19] and the closely related references therein.

From (1.2) and (1.3), it is easy to see that $m_{n}^{(u,l,d)}=m_{n}^{(d,l,u)}$. Comparing (1.2) with (1.4) reveals that $M_{k}(a,b)$ and $m_{k}^{(u,l,d)}$ are equivalent to each other and satisfy

1.6 $$ M_{k}(a,b)=m_{n}^{(1,a,b)}=m_{k}^{(b,a,1)} \quad\mbox{and}\quad m_{k}^{(u,l,d)}=M_{k}(l,ud). $$

Therefore, it suffices to consider the generalized Motzkin numbers $M_{k}(a,b)$, rather than the $(u,l,d)$-Motzkin numbers $m_{n}^{(u,l,d)}$, in this paper.

The main aim of this paper is to establish explicit formulas for the Motzkin numbers $M_{k}$ and the generalized Motzkin numbers $M_{k}(a,b)$. As consequences, two explicit formulas for the restricted hexagonal numbers $H_{n}$ are derived.

Our main results in this paper can be stated as the following theorems.

### Theorem 1

*For* $k\ge0$, *the Motzkin numbers* $M_{k}$ *can be computed by*

1.7 $$ M_{k}=\frac{9}{8} \biggl(\frac{3}{2} \biggr)^{k}\sum_{\ell=0}^{k+2} \biggl( \frac{2}{3} \biggr)^{\ell}\frac{(2\ell-3)!!}{\ell!}\binom{\ell}{k-\ell+2}, $$

*where* $\binom{p}{q}=0$ *for* $q>p\ge0$ *and the double factorial of negative odd integers* $-(2n+1)$ *is defined by*

$$ \bigl[-(2n+1)\bigr]!!=\frac{(-1)^{n}}{(2n-1)!!}=(-1)^{n}\frac{2^{n}n!}{(2n)!}, \quad n=0,1,\ldots. $$

### Theorem 2

*For* $k\ge0$ *and* $a,b\in\mathbb{N}$, *the generalized Motzkin numbers* $M_{k}(a,b)$ *can be computed by*

1.8 $$ M_{k}(a,b)=\frac{1}{2b} \biggl(\frac{4b-a^{2}}{2a} \biggr)^{k+2} \sum_{\ell =0}^{k+2} \biggl(\frac{2a^{2}}{4b-a^{2}} \biggr)^{\ell}\frac{(2\ell-3)!!}{\ell !} \binom{\ell}{k-\ell+2}. $$

*Consequently*, *the Catalan numbers* $C_{k}$ *and the restricted hexagonal numbers* $H_{k}$ *can be computed by*

1.9 $$ C_{k}=2^{k}\frac{(2k-1)!!}{(k+1)!} $$

*and*

1.10 $$ H_{k}=(-1)^{k}\frac{25}{72} \biggl( \frac{5}{6} \biggr)^{k} \sum_{\ell=0}^{k+2} (-1)^{\ell}\biggl(\frac{18}{5} \biggr)^{\ell}\frac{(2\ell-3)!!}{\ell!}\binom {\ell}{k-\ell+2}, $$

*respectively*.

### Theorem 3

*For* $n\ge0$ *and* $a,b\in\mathbb{N}$, *the generalized Motzkin numbers* $M_{n}(a,b)$ *can be computed by*

1.11 $$ M_{n}(a,b)= \textstyle\begin{cases} 1, & n=0;\\ \displaystyle\frac{a^{2}}{2b} \biggl(\frac{4b-a^{2}}{2a} \biggr)^{n} \sum_{k=0}^{n} \biggl(\frac{2a^{2}}{4b-a^{2}} \biggr)^{k} \frac{(2k+1)!!}{(k+2)!} \binom {k+2}{n-k}, & n\in\mathbb{N}. \end{cases} $$

*Consequently*, *equation* (1.9) *for the Catalan numbers* $C_{n}$ *is valid*, *the Motzkin numbers* $M_{n}$ *and the restricted hexagonal numbers* $H_{k}$ *can be computed by*

1.12 $$ M_{n}= \textstyle\begin{cases} 1, & n=0,\\ \displaystyle\frac{1}{2} \biggl(\frac{3}{2} \biggr)^{n} \sum_{k=0}^{n} \biggl(\frac{2}{3} \biggr)^{k} \frac{(2k+1)!!}{(k+2)!} \binom{k+2}{n-k}, & n\in\mathbb{N,} \end{cases} $$

*and*

1.13 $$ H_{n}= \textstyle\begin{cases} 1, & n=0,\\ \displaystyle(-1)^{n}\frac{9}{2} \biggl(\frac{5}{6} \biggr)^{n} \sum_{k=0}^{n} (-1)^{k} \biggl(\frac{18}{5} \biggr)^{k} \frac{(2k+1)!!}{(k+2)!} \binom{k+2}{n-k}, & n\in \mathbb{N,} \end{cases} $$

*respectively*.

## Proofs of main results

Now we are in a position to prove our main results.

### Proof of Theorem 1

From (1.1), it follows that

$$ \sqrt{1-2x-3x^{2}} =1-x-2\sum_{k=0}^{\infty}M_{k}x^{k+2}. $$

This implies that

2.1 $$ M_{k}=-\frac{1}{2}\frac{1}{(k+2)!}\lim _{x\to0} \bigl(\sqrt{1-2x-3x^{2}} \bigr)^{(k+2)}, \quad k\ge0. $$

In combinatorial analysis, the Faà di Bruno formula plays an important role and can be described in terms of the Bell polynomials of the second kind

$$ \mathrm {B}_{n,k}(x_{1},x_{2},\dotsc,x_{n-k+1})= \sum_{\substack{1\le i\le n,\ell _{i}\in\{0\}\cup\mathbb{N}\\ \sum_{i=1}^{n}i\ell_{i}=n\\ \sum_{i=1}^{n}\ell_{i}=k}}\frac{n!}{\prod_{i=1}^{n-k+1}\ell_{i}!} \prod _{i=1}^{n-k+1} \biggl(\frac{x_{i}}{i!} \biggr)^{\ell_{i}} $$

for $n\ge k\ge0$, see [20], p.134, Theorem A, by

2.2 $$ \frac{\mathrm {d}^{n}}{\mathrm {d}t^{n}}\bigl[f\circ h(t)\bigr]=\sum _{k=0}^{n}f^{(k)}\bigl(h(t)\bigr) \mathrm {B}_{n,k} \bigl(h'(t),h''(t), \dotsc,h^{(n-k+1)}(t) \bigr) $$

for $n\ge0$; see [20], p.139, Theorem C. The Bell polynomials of the second kind $\mathrm {B}_{n,k}(x_{1},x_{2}, \ldots, x_{n-k+1})$ satisfy the formula

2.3 $$ \mathrm {B}_{n,k} \bigl(abx_{1},ab^{2}x_{2}, \dotsc,ab^{n-k+1}x_{n-k+1} \bigr) =a^{k}b^{n} \mathrm {B}_{n,k}(x_{1},x_{2},\dotsc,x_{n-k+1}) $$

for $n\ge k\ge0$; see [20], p.135. In [21], Theorem 4.1, [10], Eq. (2.8), and [22], Section 3, it was established that

2.4 $$ \mathrm {B}_{n,k}(x,1,0,\ldots,0) =\frac{(n-k)!}{2^{n-k}} \binom{n}{k}\binom{k}{n-k}x^{2k-n}, \quad n\ge k\ge0. $$

Then, for $k\ge0$, we have

$$\begin{aligned} \bigl(\sqrt{1-2x-3x^{2}} \bigr)^{(k+2)} &=\sum _{\ell=0}^{k+2} \biggl\langle \frac{1}{2} \biggr\rangle _{\ell} \bigl(1-2x-3x^{2} \bigr)^{1/2-\ell} \mathrm {B}_{k+2,\ell}(-2-6x,-6,0,\dotsc,0) \\ &\to \sum_{\ell=0}^{k+2} \biggl\langle \frac{1}{2} \biggr\rangle _{\ell} \mathrm {B}_{k+2,\ell}(-2,-6,0, \dotsc,0) \\ &=\sum_{\ell=0}^{k+2} \biggl\langle \frac{1}{2} \biggr\rangle _{\ell} (-6)^{\ell} \mathrm {B}_{k+2,\ell} \biggl(\frac{1}{3},1,0,\dotsc,0 \biggr) \\ &=\sum_{\ell=0}^{k+2}(-1)^{\ell}\biggl\langle \frac{1}{2} \biggr\rangle _{\ell} \frac{(k-\ell+2)!}{2^{k-2\ell+2}3^{\ell-k-2}} \binom{k+2}{\ell}\binom {\ell}{k-\ell+2} \end{aligned}$$

as $x\to0$, where

$$ \langle x\rangle_{n}= \textstyle\begin{cases} x(x-1)\dotsm(x-n+1), & n\ge1,\\ 1,& n=0, \end{cases} $$

denotes the falling factorial of $x\in\mathbb{R}$. Consequently, by (2.1), it follows that

$$ M_{k}=-\frac{9}{8} \biggl(\frac{3}{2} \biggr)^{k}\frac{1}{(k+2)!}\sum_{\ell=0}^{k+2} (-1)^{\ell}\biggl\langle \frac{1}{2} \biggr\rangle _{\ell} \biggl(\frac{4}{3} \biggr)^{\ell}(k-\ell+2)!\binom{k+2}{\ell} \binom{\ell }{k-\ell+2} $$

for $k\ge0$, which can be rewritten as (1.7). The proof of Theorem 1 is complete. □

### Proof of Theorem 2

From (1.4), it is derived that

$$ \sqrt{(1-ax)^{2}-4bx^{2}} =1-ax-2b\sum _{k=0}^{\infty}M_{k}(a,b)x^{k+2}. $$

This implies that

2.5 $$ M_{k}(a,b)=-\frac{1}{2b}\frac{1}{(k+2)!}\lim _{x\to0} \bigl[\sqrt {(1-ax)^{2}-4bx^{2}} \bigr]^{(k+2)}, \quad k\ge0. $$

By virtue of (2.2), (2.3), and (2.4), it follows that

$$\begin{aligned} & \bigl[\sqrt{(1-ax)^{2}-4bx^{2}} \bigr]^{(k+2)} \\ &\quad=\sum_{\ell=0}^{k+2} \biggl\langle \frac{1}{2} \biggr\rangle _{\ell} \bigl[(1-ax)^{2}-4bx^{2} \bigr]^{1/2-\ell} \\ &\qquad{}\times \mathrm {B}_{k+2,\ell} \bigl(-2 \bigl[a+ \bigl(4b-a^{2} \bigr)x \bigr], 2 \bigl(a^{2}-4b \bigr),0,\dotsc,0 \bigr) \\ &\quad\to\sum_{\ell=0}^{k+2} \biggl\langle \frac{1}{2} \biggr\rangle _{\ell} \mathrm {B}_{k+2,\ell} \bigl(-2a,2 \bigl(a^{2}-4b \bigr),0,\dotsc,0 \bigr) \\ &\quad=\sum_{\ell=0}^{k+2} \biggl\langle \frac{1}{2} \biggr\rangle _{\ell} \bigl[2 \bigl(a^{2}-4b \bigr) \bigr]^{\ell} \mathrm {B}_{k+2,\ell} \biggl(\frac {a}{4b-a^{2}},1,0, \dotsc,0 \biggr) \\ &\quad=\sum_{\ell=0}^{k+2} \biggl\langle \frac{1}{2} \biggr\rangle _{\ell} \bigl[2 \bigl(a^{2}-4b \bigr) \bigr]^{\ell}\frac{(k-\ell+2)!}{2^{k-\ell+2}} \binom{k+2}{\ell} \binom{\ell}{k-\ell+2} \biggl(\frac{a}{4b-a^{2}} \biggr)^{2\ell-k-2} \end{aligned}$$

as $x\to0$. Substituting this into (2.5) and simplifying yield

$$ M_{k}(a,b)=-\frac{1}{2b}\sum_{\ell=0}^{k+2} \biggl\langle \frac{1}{2} \biggr\rangle _{\ell} \bigl[2 \bigl(a^{2}-4b \bigr) \bigr]^{\ell}\frac{1}{2^{k-\ell+2}} \frac{1}{\ell !}\binom{\ell}{k-\ell+2} \biggl(\frac{a}{4b-a^{2}} \biggr)^{2\ell-k-2} $$

for $k\ge0$, which can be further rearranged as (1.8).

Letting $(a,b)=(2,1)$ and $(a,b)=(3,1)$, respectively, in (1.8) and considering the last two relations in (1.5) lead to (1.9) and (1.10) immediately. The proof of Theorem 2 is complete. □

### Proof of Theorem 3

For $|x [ (a^{2}-4b )x-2a ] |<1$, the generating function $M_{a,b}(x)$ in (1.4) can be expanded into

$$\begin{aligned} M_{a,b}(x)&=\frac{1}{2bx^{2}} \bigl[1-ax-\sqrt{1-2ax+ \bigl(a^{2}-4b\bigr)x^{2}} \bigr] \\ &=\frac{1}{2bx^{2}} \Biggl\{ 1-ax-\sum_{k=0}^{\infty}\biggl\langle \frac{1}{2} \biggr\rangle _{k} \frac{x^{k} [ (a^{2}-4b )x-2a ]^{k}}{k!} \Biggr\} \\ &=\frac{1}{2bx^{2}} \Biggl\{ -\frac{a^{2}-4b}{2}x^{2}-\sum _{k=2}^{\infty}\biggl\langle \frac{1}{2} \biggr\rangle _{k} \frac{x^{k} [ (a^{2}-4b )x-2a ]^{k}}{k!} \Biggr\} \\ &=-\frac{1}{2b} \Biggl\{ \frac{a^{2}-4b}{2}+\sum _{k=2}^{\infty}\biggl\langle \frac{1}{2} \biggr\rangle _{k} \frac{x^{k-2} [ (a^{2}-4b )x-2a ]^{k}}{k!} \Biggr\} . \end{aligned}$$

By (1.4) once again, it follows that

$$\begin{aligned} M_{n}(a,b)&=\frac{1}{n!}\lim_{x\to0} \bigl[M_{a,b}(x)\bigr]^{(n)} \\ &=-\frac{1}{2b}\frac{1}{n!}\lim_{x\to0} \Biggl\{ \frac{a^{2}-4b}{2} +\sum_{k=2}^{\infty}\biggl\langle \frac{1}{2} \biggr\rangle _{k} \frac{x^{k-2} [ (a^{2}-4b )x-2a ]^{k}}{k!} \Biggr\} ^{(n)}, \end{aligned}$$

which means that

$$\begin{aligned} M_{0}(a,b)&=-\frac{1}{2b}\lim_{x\to0} \Biggl\{ \frac{a^{2}-4b}{2} +\sum_{k=2}^{\infty}\biggl\langle \frac{1}{2} \biggr\rangle _{k} \frac{x^{k-2} [ (a^{2}-4b )x-2a ]^{k}}{k!} \Biggr\} \\ &=-\frac{1}{2b} \biggl[\frac{a^{2}-4b}{2} + \biggl\langle \frac{1}{2} \biggr\rangle _{2} \frac{4a^{2}}{2!} \biggr] \\ &=1 \end{aligned}$$

and

$$\begin{aligned} M_{n}(a,b)={}&{-}\frac{1}{2b}\frac{1}{n!}\lim _{x\to0}\sum_{k=2}^{\infty}\biggl\langle \frac{1}{2} \biggr\rangle _{k} \frac{ \{x^{k-2} [ (a^{2}-4b )x-2a ]^{k} \}^{(n)}}{k!} \\ ={}&{-}\frac{1}{2b}\frac{1}{n!}\lim_{x\to0}\sum _{k=0}^{\infty}\biggl\langle \frac{1}{2} \biggr\rangle _{k+2} \frac{ \{x^{k} [ (a^{2}-4b )x-2a ]^{k+2} \}^{(n)}}{(k+2)!} \\ ={}&{-}\frac{1}{2b}\frac{1}{n!}\lim_{x\to0}\sum _{k=0}^{\infty}\frac{\langle 1/2\rangle_{k+2}}{(k+2)!} \\ &{}\times\Biggl\{ \sum _{\ell=0}^{k+2}\binom{k+2}{\ell} \bigl(a^{2}-4b \bigr)^{\ell}(-2a)^{k-\ell+2} x^{k+\ell} \Biggr\} ^{(n)} \\ ={}&{-}\frac{1}{2b}\frac{1}{n!}\lim_{x\to0}\sum _{k=0}^{\infty}\frac{\langle 1/2\rangle_{k+2}}{(k+2)!} \sum _{\ell=0}^{k+2}\binom{k+2}{\ell} \bigl(a^{2}-4b \bigr)^{\ell}(-2a)^{k-\ell+2} \bigl(x^{k+\ell} \bigr)^{(n)} \\ ={}&\frac{1}{2b}\lim_{x\to0}\sum _{k=0}^{\infty}\frac{(2k+1)!!}{(k+2)!} \sum _{\ell=n-k}^{k+2}\binom{k+2}{\ell} \biggl( \frac{4b-a^{2}}{2} \biggr)^{\ell}a^{k-\ell+2} \binom{k+\ell}{n}x^{k+\ell-n} \\ ={}&\frac{1}{2b}\sum_{k=0}^{n} \frac{(2k+1)!!}{(k+2)!} \binom{k+2}{n-k} \biggl(\frac{4b-a^{2}}{2} \biggr)^{n-k} a^{2k-n+2} \\ ={}&\frac{a^{2}}{2b} \biggl(\frac{4b-a^{2}}{2a} \biggr)^{n} \sum _{k=0}^{n}\frac {(2k+1)!!}{(k+2)!} \binom{k+2}{n-k} \biggl(\frac{2a^{2}}{4b-a^{2}} \biggr)^{k} \end{aligned}$$

for $n\in\mathbb{N}$. In conclusion, equation (1.11) follows.

Taking $(a,b)=(2,1)$, $(a,b)=(1,1)$, and $(a,b)=(3,1)$, respectively, in (1.11) and considering the three relations in (1.5) lead to (1.9), (1.12), and (1.13) readily. The proof of Theorem 3 is complete. □

## Remarks

Finally, we list several remarks.

### Remark 1

The explicit formula (1.8) is a generalization of (1.7).

### Remark 2

Equation (1.9) and many other alternative formulas for the Catalan numbers $C_{k}$ can also be found in [3–6, 8, 9, 12–14] and the closely related references therein.

### Remark 3

By the second relation in (1.6), equation (1.3) can be reformulated as

3.1 $$ M_{n}(a,b)=a^{n}\sum _{j=0}^{n/2}\frac{1}{j+1}\binom{2j}{j} \binom{n}{2j} \biggl(\frac{b}{a^{2}} \biggr)^{j}, $$

which is different from the two equations (1.8) and (1.11).

### Remark 4

Making use of any one among equations (1.8), (1.11), and (3.1), we can present the first nine generalized Motzkin numbers $M_{n}(a,b)$ for $0\le n\le8$ and $a,b\in \mathbb{N}$ as follows:

$$\begin{aligned}& 1, \quad a,\quad a^{2}+b, \quad a \bigl(a^{2}+3 b \bigr), \quad a^{4}+6 a^{2} b+2 b^{2},\quad a \bigl(a^{4}+10 a^{2} b+10 b^{2} \bigr), \\& a^{6}+15 a^{4} b+30 a^{2} b^{2}+5 b^{3}, \quad a \bigl(a^{6}+21 a^{4} b+70 a^{2} b^{2}+35 b^{3} \bigr), \\& a^{8}+28 a^{6} b+140 a^{4} b^{2}+140 a^{2} b^{3}+14 b^{4}. \end{aligned}$$

In particular, the first nine restricted hexagonal numbers $H_{n}$ for $0\le n\le8$ are

$$ 1,\quad 3,\quad 10,\quad 36,\quad 137,\quad 543,\quad 2{,}219,\quad 9{,}285, \quad 39{,}587. $$

## Conclusions

By the Faà di Bruno formula and some properties of the Bell polynomials of the second kind, we establish two explicit formulas for the Motzkin numbers, the generalized Motzkin numbers, and the restricted hexagonal numbers.
